# Supplementary material for: Immune Memory in Mild COVID-19 Patients and Unexposed Donors Reveals Persistent T Cell Responses After SARS-CoV-2 Infection
Source: Front Immunol. 2021 Mar 11;12:636768. doi: 10.3389/fimmu.2021.636768 (PMC7991090; doi:10.3389/fimmu.2021.636768)
Supplement: Supplementary file 1 [file DataSheet_1.pdf]

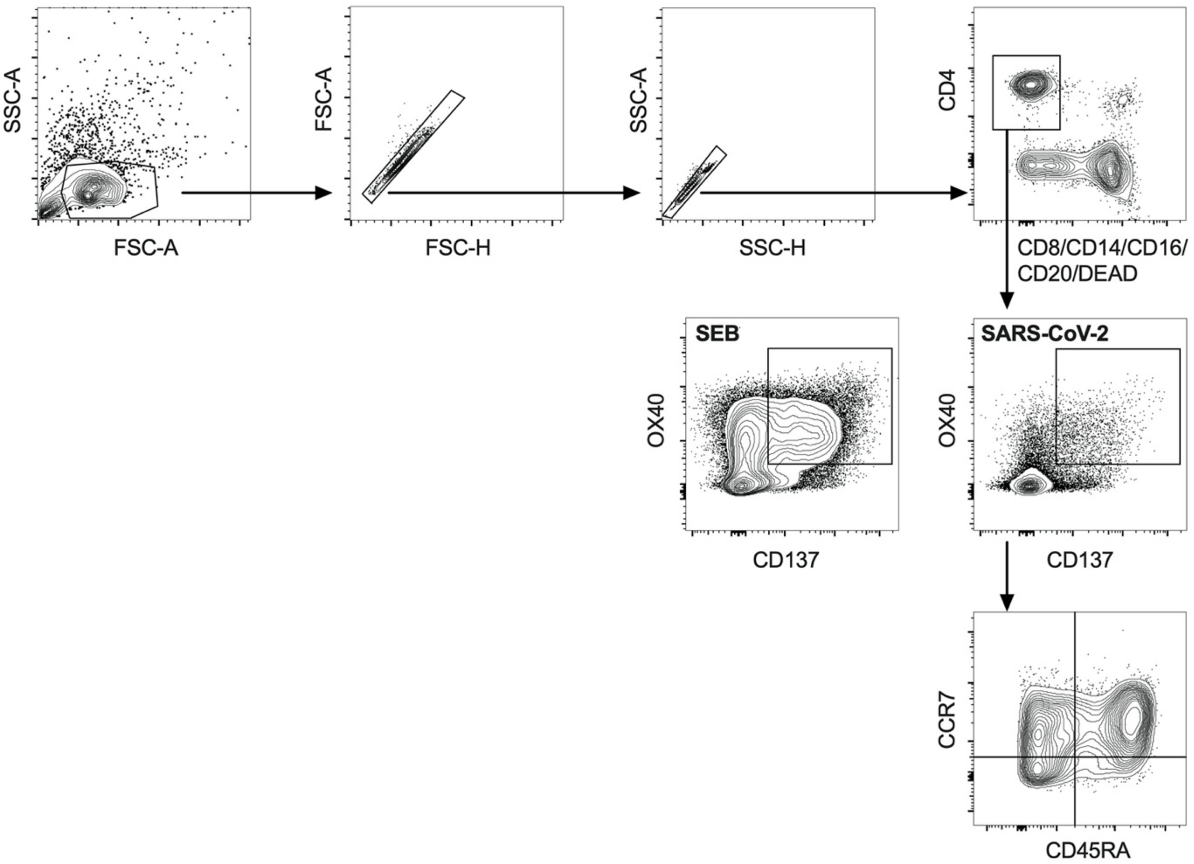

Supplementary Figure 1. Flow cytometry Gating Strategy. The example gating strategy for analysing the OX40^{+}CD137^{+} (AIM^{+}) CD4^{+} T cells in the PBMCs from unexposed donors and the recovered COVID-19 patients.

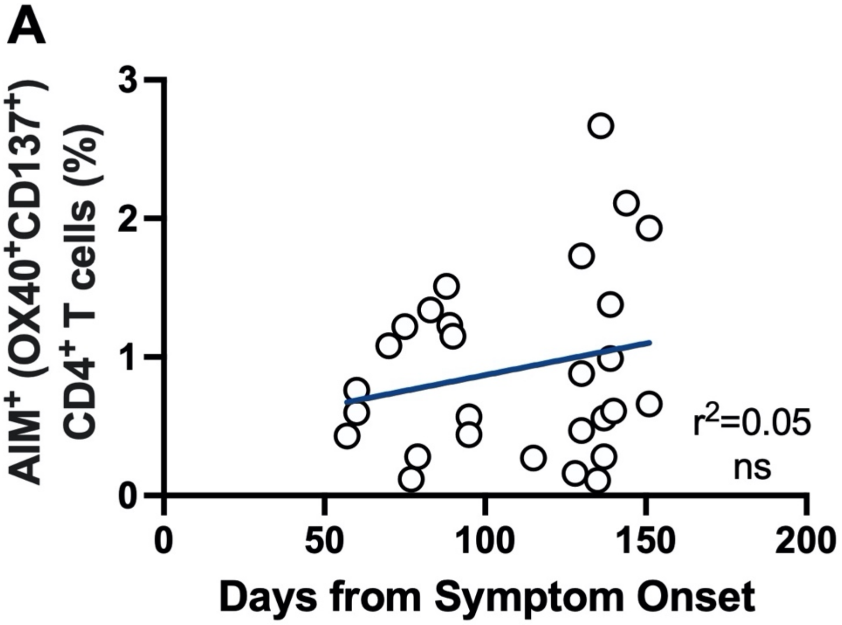

Supplementary Figure 2. Correlation of SARS-CoV-2 Spike-specific CD4$^+$ T cell response with the time from symptoms onset. The frequency of SARS-CoV-2 specific CD4$^+$ T cells was determined in the subjects recovered from COVID-19. The PBMCs were stimulated with the peptide megapool specific to Spike glycoprotein (Spike) and antigen-specific cells were determined using the AIM assay. (**A**) Correlation of the frequency of AIM$^+$ Spike-specific CD4$^+$ T cells with the days from symptoms onset in recovered patients. Each circle represents a COVID-19 subject. Statistical analysis was performed by simple linear regression. ns: non-significant.

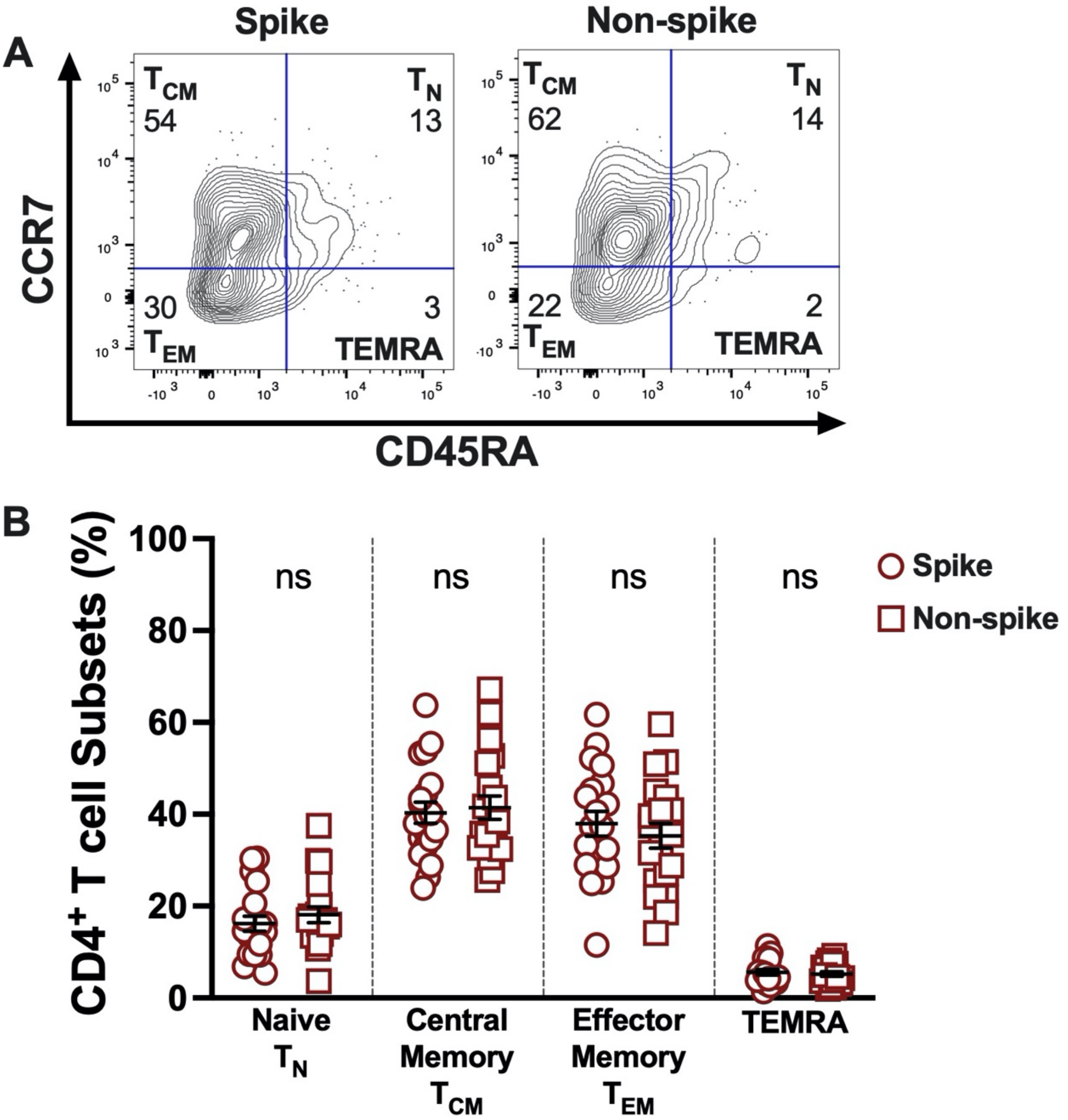

Supplementary Figure 3. Memory phenotype of SARS-CoV-2-specific $CD4^{+}$ T cells in recovered COVID-19 patients. The memory phenotype of the antigen-specific $CD4^{+}$ T cells was determined in the COVID-19 recovered subjects (n=20). The PBMCs were stimulated with the peptide megapool specific to Spike glycoprotein (Spike) or to the remainder of the SARS-CoV-2 polyprotein (Non-spike). (**A**) Example FACS plot for analysing the memory phenotype on antigen-specific AIM^{+} ($OX40^{+}CD137^{+}$) $CD4^{+}$ T cells. (**B**) Proportion of memory phenotype in the antigen-specific $AIM^{+}CD4^{+}$ T cells. Phenotypes of $AIM^{+}CD4^{+}$ T cells were defined by the expression of CD45RA and CCR7. $CD4^{+}$ T cell subsets were defined as naïve (T_N) cells ($CD45RA^{+}CCR7^{+}$), central memory ($T_{CM)}$ cells ($CD45RA^{-}CCR7^{+}$), effector memory (T_{EM}) cells ($CD45RA^{-}CCR7^{-}$), and TEMRA cells ($CD45RA^{+}CCR7^{-}$). Black bars indicate the geometric mean. Statistical comparisons were performed by two-tail Mann-Whitney test. ns: non-significant.

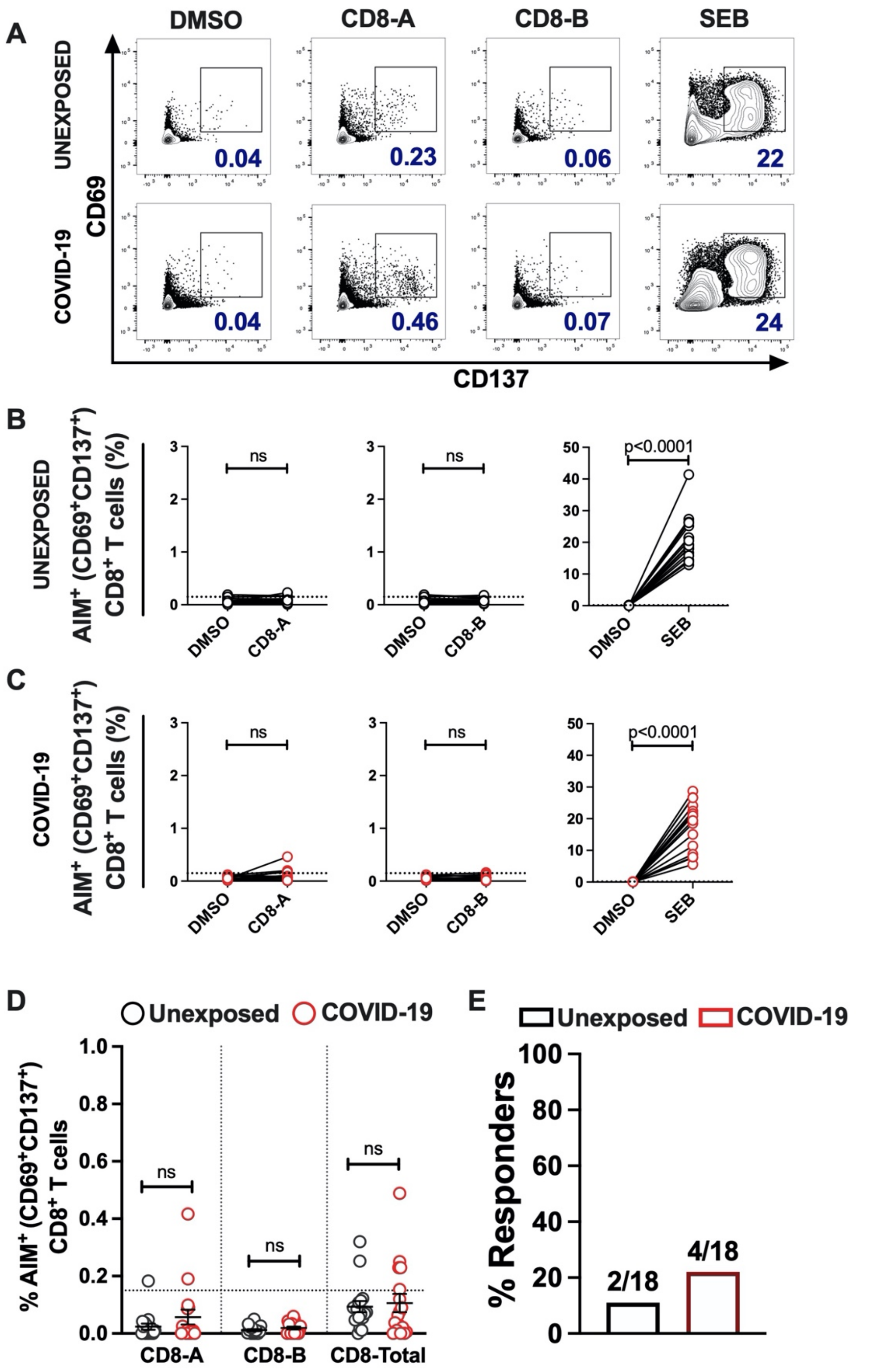

Supplementary Figure 4. SARS-CoV-2-specific CD8^{+} T cells response in unexposed donors and recovered COVID-19 patients. The magnitude of SARS-CoV-2 specific CD8^{+} T cells was determined in PBMCs collected from unexposed donors ("Unexposed", n=18) prior to pandemic and in COVID-19 patients ("COVID-19", n=18) up to 5 months of recovery. The PBMCs were stimulated with the class I peptide megapool (MP) comprises of 628 predicted CD8 epitopes and split into two MPs (CD8-A and CD8-B). DMSO was used as the negative control, and SEB was used for positive stimulation control. **(A)** Representative FACS contour plots of unexposed and COVID-19 patient in stimulation conditions of DMSO, CD8-A MP, CD8-B MP, and SEB. Paired graphs depicting the reactivity of AIM^{+} (CD69^{+}CD137^{+}) CD8^{+}

T cells between the negative control (DMSO) and antigen-specific stimulation in (**B**) Unexposed donors (**C**) COVID-19 patients. **(D)** Percentage of the SARS-CoV-2 specific AIM^{+} ($CD69^{+}CD137^{+}$) $CD8^{+}$ T cells after stimulation with the class I MPs (CD8-A, CD8-B) and the combined data of both the MPs, analysed in the same samples as in panel B and C. The data obtained in MPs stimulation were background subtracted with the respective DMSO negative controls. Black bars indicate the geometric mean. **(E)** Frequency of responders in unexposed and COVID-19 recovered subjects determined from the combined data of CD8-A and CD8-B MPs after background subtracted from the DMSO negative controls. The value on bars denote the number of responders/total number of donors tested. Dotted line in panels B, C and D represent the limit of detection. Statistical comparisons were performed by (B-C) Wilcoxon paired t-test and (D) two-tail Mann-Whitney test. ns: non-significant.
